# Trick or Treat? Licorice-Induced Hypokalemia: A Case Report

**DOI:** 10.7759/cureus.11656

**Published:** 2020-11-23

**Authors:** Elizabeth Benge, Pinak Shah, Leo Yamaguchi, Vanessa Josef

**Affiliations:** 1 Internal Medicine, Mountainview Hospital, Las Vegas, USA

**Keywords:** apparent mineralocorticoid-excess, pseudohyperaldosteronism, hypokalemia, black licorice

## Abstract

The by-products of black licorice metabolism are toxic in high concentrations. Patients who consume large quantities of black licorice are at risk of developing an acquired syndrome of apparent mineralocorticoid excess. This presents clinically as hypertension, hypernatremia, and hypokalemia. Here, we present the unique case of a 74-year-old woman with a past medical history of neurogenic orthostatic hypotension, on fludrocortisone, who presented to the emergency department with asymptomatic hypokalemia (2.4 mmol/L) as detected in outpatient laboratory studies. During her hospital stay, it was discovered that the patient was consuming excessive amounts of black licorice. With this information, the synergistic interaction of fludrocortisone and black licorice was recognized as the cause of the patient’s severe hypokalemia. The patient’s fludrocortisone was stopped and she was treated with multiple courses of potassium repletion. Upon discharge, her fludrocortisone was discontinued, and she was prescribed midodrine to treat her neurogenic orthostatic hypertension. While small amounts of black licorice are safe, excessive licorice consumption can cause severe disease. Our case presents an opportunity to appreciate the plethora of etiologies for severe hypokalemia and the importance of taking a thorough patient history to avoid potentially fatal clinical outcomes.

## Introduction

As early as 2100 BC, practitioners of traditional medicine used licorice to treat maladies ranging from pain and cough, to gastrointestinal illness [1]. Unfortunately, the bioactive properties of licorice can pose a threat to modern patients [2]. The by-products of licorice metabolism inhibit renal 11-ß-hydroxysteroid dehydrogenase (11β-HSD2) activity, decreasing the conversion of cortisol to the inactive form [3]. Consequently, endogenous plasma cortisol is available to bind to mineralocorticoid receptors for a longer period of time, creating a syndrome known as apparent mineralocorticoid excess (AME) or pseudohyperaldosteronism [3]. Thus, patients who eat licorice regularly can experience hypertension, hypernatremia, and hypokalemia [4]. Patients at risk for severe symptoms include the elderly, women, or those with pre-existing conditions such as hypokalemia, hypertension, and anorexia nervosa [5]. Here, we present a unique case of an elderly, female patient who consumed black licorice while taking fludrocortisone and subsequently developed severe hypokalemia, due to their synergistic combination.

## Case presentation

A 74-year-old woman with a significant past medical history of Parkinson’s disease (on carbidopa/levodopa) and neurogenic orthostatic hypotension presented to the emergency department for hypokalemia as directed by her outpatient provider. On presentation, the patient denied experiencing symptoms associated with hypokalemia, including weakness, fatigue, vomiting, abdominal pain, cramping and tingling in her extremities. She stated that she felt at her baseline state of health and a 12-point review of systems was negative. When reviewing her medications, she reported taking 0.1 mg of fludrocortisone twice a day at home regularly without any missed doses. The patient’s vital signs were all within normal limits upon her arrival to the hospital, with a blood pressure of 102/70 mmHg. Her physical exam did not reveal any signs of acute pathology. 

Her initial lab values demonstrated severe hypokalemia, with a potassium level of 2.4 mmol/L. Her arterial blood gas revealed metabolic alkalosis. The remainder of her initial blood work, which included a complete metabolic panel and a complete blood count, was normal. The patient’s electrocardiogram showed a ventricular-paced rhythm with a heart rate of 82 beats/min without ST-segment elevations, heart blocks, or axis deviations. Her fludrocortisone was held at that time and her potassium was repleted with both 40 meq of oral potassium and 20 meq of intravenous potassium chloride. She was subsequently admitted to the hospital for further evaluation and management of her severe hypokalemia.

Upon admission, the patient’s hypokalemia was thought to be caused by her fludrocortisone, with a secondary differential diagnosis of adrenal insufficiency. Urine electrolytes, urine creatinine, urine osmolality, thyroid panel, and plasma cortisol lab studies were ordered to further elucidate the cause of the patient’s disease.

A follow-up potassium level, drawn two hours after the patient’s potassium had been repleted, was 2.4 mmol/L, unchanged from her initial level. Her potassium was again repleted with both IV and oral potassium. Her blood pressure increased to 151/77 mmHg at 8:00 am, five hours after she was admitted. It then increased to 171/82 mmHg at 11:00 am. At that time, her hypertension was treated with 5 mg of IV hydralazine, and her blood pressure decreased to 109/65 mmHg over the next several hours. The patient’s follow-up lab results showed a low plasma renin activity at <0.167 ng/mL/hr and a low aldosterone level at <1.0 ng/dL. Notably, her plasma cortisol level was normal at 11.20 mcg/dL. Her urine studies and thyroid panel results did not reveal any signs of disease.

The morning after the patient’s admission, she informed the internal medicine team that she had recently been eating large quantities of black licorice. This information, alongside the patient’s lab results, narrowed the differential diagnosis. A synergistic interaction between licorice and the fludrocortisone was now suspected as the cause of the patient’s hypokalemia. The endocrinology service was consulted and agreed with the primary team’s assessment. They recommended stopping the fludrocortisone and repleting potassium as needed.

The patient’s potassium level increased to a normal value of 3.5 mmol/L five hours after her second course of potassium repletion, roughly thirty-four hours after her last dose of fludrocortisone. She required two more administrations of 5 mg IV hydralazine to keep her systolic blood pressure below 150 mmHg. 

She remained in the hospital for another night for continued observation and monitoring of her potassium. At the time of discharge, the patient’s potassium was 3.5 mmol/L, and her metabolic alkalosis had resolved. She was instructed to stop taking fludrocortisone at home, and was prescribed midodrine to treat her neurogenic orthostatic hypotension. She was informed about the dangers of combining licorice and fludrocortisone and asked to follow-up with her primary care provider for further management.

## Discussion

The renin‐angiotensin‐aldosterone system (RAAS) is the hormonal feedback loop responsible for regulating sodium and potassium homeostasis and fluid balance [6]. Normally, renin is released from the kidney in low-blood volume states, and its downstream effects work to maintain healthy blood pressure. One such action of renin is that it increases plasma aldosterone [7]. Aldosterone works to decrease serum potassium, increase serum sodium, and ultimately increase blood pressure [8]. 

Our patient’s lab values showed low renin, low aldosterone, and severe hypokalemia, along with normal plasma cortisol levels and normal urine studies. She was also intermittently hypertensive. 

Importantly, our patient had also been taking fludrocortisone, a steroid medication with a similar mechanism of action to endogenous aldosterone [9]. Fludrocortisone potently decreases serum potassium levels, and can be used to treat hyperkalemia [9]. This medication contributed to the severity of our patient’s electrolyte imbalance. 

Thus, the differential diagnoses for our patient revolved around etiologies of low-renin hypertension, such as Conn syndrome, Liddle's syndrome, deficiencies of enzymes 11β-hydroxylase or 17α-hydroxylase, and AME [10]. 

Conn Syndrome is a disorder characterized by primary hyperaldosteronism due to an aldosterone-secreting adenoma in the adrenal gland [11]. The autonomous secretion of aldosterone suppresses plasma renin activity, resulting in low plasma renin activity. Thus, Conn syndrome is associated with an increase in plasma aldosterone, which excludes this syndrome as the cause of our patient’s disease. 

Deficiencies of enzymes 11β-hydroxylase and 17α-hydroxylase are examples of congenital adrenal hyperplasias. They are caused by inherited defects in CYP11B1 and CYP17 genes, which encode the corresponding enzymes, and are passed down in an autosomal recessive pattern [12]. Both conditions result in a decreased serum cortisol level. Deficiency of 17-alpha hydroxylase is accompanied by an increase in mineralocorticoids, while 11-beta hydroxylase deficiency is characterized by decreased aldosterone levels and a decrease in plasma renin activity [12]. While these enzyme deficiencies have a clinical presentation and several lab values consistent with our patient’s presentation, her plasma cortisol level was normal, while the hallmark of these diseases is low cortisol. Additionally, the age of onset would be highly unusual for these genetic diseases. 

Patients with Liddle's syndrome present with hypertension due to inherited mutations in the epithelial Na+ channel (ENaC) in the collecting tubules of the kidney [13]. This disease is characterized by abnormal urine studies: increased urinary potassium excretion, decreased urinary sodium excretion, low rate of urinary aldosterone excretion, and a low ratio of aldosterone to potassium [14]. Clinically, Liddle’s syndrome mimics hyperaldosteronism, however, serum plasma aldosterone levels are severely decreased [14]. Our patient’s normal urine studies made Liddle’s syndrome an unlikely culprit. 

AME is a genetic disease that results from the inability of the enzyme 11β-hydroxysteroid dehydrogenase to convert cortisol to inactive cortisone [15]. With this enzyme malfunctioning, plasma cortisol is able to bind to the mineralocorticoid receptor with the same affinity as aldosterone, while circulating with a magnitude of 1000-2000 times greater than aldosterone [16]. This results in a clinical presentation of hypertension and hypokalemia at a young age [17]. Additionally, due to feedback inhibition, the endogenous renin-angiotensin-aldosterone system is suppressed, which leads to low renin and aldosterone levels [17].

Licorice produces symptoms that mirror AME, and should also be considered as a differential diagnosis (Table 1). Glycyrrhizic acid, a by-product of licorice metabolism, inhibits the peripheral conversion of cortisol to the inactive cortisone [18]. This results in a hypermineralocorticoid clinical presentation, as cortisol binds to mineralocorticoid receptors with the same affinity as aldosterone, but is present in the bloodstream at a far higher concentration compared to aldosterone [18]. Thus, given our patient’s age of presentation, lab values, and licorice consumption, her presentation was most consistent with hypertension with an etiology of an AME caused by chronic licorice ingestion and exacerbated by her simultaneous use of steroid medications [19].

**Table 1:** Differential Diagnosis

Disease	Serum Potassium	Plasma Renin Activity	Plasma Aldosterone Levels	Plasma Cortisol Levels
Conn Syndrome	↓ / Normal	↓	↑	Normal
11β-hydroxylase deficiency	↓	↓	↓	↓
Apparent Mineralocorticoid Excess	↓	↓	↓	Normal

Due to the rarity of our patient’s condition, there is little evidence-based medicine to guide the management of her multifactorial etiology of hypertension. The first steps we took in her treatment course were to treat her severe hypokalemia with potassium supplementation and discontinue her mineralocorticoid medication. We monitored her potassium closely while the etiology of her hypokalemia was being investigated. On discharge, we discontinued her steroid medication and prescribed her an alternative treatment for her neurogenic hypotension. We also provided our patient with extensive education about the dangers of consuming licorice in such high quantities. 

Prior documented cases of licorice-induced hypertension have been successfully treated with mineralocorticoid receptor antagonists (ex. spironolactone) and other potassium-sparing diuretics (ex. amiloride) [20]. After large quantity licorice consumption, RAAS may remain suppressed for as long as three months. Thus, follow-up electrolyte studies and close monitoring of blood pressure is recommended [20].

This article was previously presented as a virtual poster at the 2020 Nevada American College of Physicians Chapter Abstracts Competition-Southern Region on October 20, 2020.

## Conclusions

The biggest takeaway lesson from our case is how important it is to take a detailed patient history. The etiology of our patient’s disease hinged on one specific, seemingly insignificant detail about her diet. It is essential that health care providers take the time to thoroughly discuss dietary habits and lifestyle choices with their patients, as it could reveal the underlying cause of their disease. Our patient’s case also presents an opportunity to appreciate the plethora of etiologies of severe hypokalemia. From medications to genetic diseases and excessive candy consumption, the sheer number of causes can be overwhelming. As illustrated in the above case, medications and diet can be a significant, even synergistic, source of severe electrolyte derangement. Although it may not be considered initially, lifestyle and diet changes are often present and may contribute to life-threatening diseases. 
